# Character and Long Life

**Published:** 1874-07

**Authors:** P. H. Hayes


### CHARACTER AND LONG LIFE.

P. H. HAYES, M. D.

There are breezes in the mental atmosphere of great men. Coming near to them, we feel the contagion of their aspirations and take a fresh interest in life.

It is not a week since the types told us that William Cullen Bryant, already highly distinguished as poet, editor and orator, and now more than eighty years old, has begun the task of writing the history of the United States, from the discovery of America to the year 1876 ! From over the sea there comes to us a still more extraordinary instance of mental energy and aspiration in old age. M. Guizot, now in his eighty-eighth year, says : "Last year I finished my history of France, and this year, please God, will see me commence my Universal History. I can hear well, see well and work well. Pius the Ninth can do the same. We are the heartiest old men in Europe ; we will outlive many that are yet young, if God please." Guizot acquired the Spanish language at seventy-two, and taught it to his grand-children. There is something in all this that stirs the blood and quickens hope in a youth of fifty; something that makes us shake off the tyrrany of the idea that we are growing old, and re-resolve the best of our life is that which we have not yet lived. After all, what is old age ? It is not *years*, for many of the most vigorous and enthusiastic workers are men full of years, who nevertheless do not seem to be *old*. Commodore Vanderbilt, the Railway King of the North, still an indomitable worker, is over eighty. A. T. Stewart at seventy-five, manages with unfailing skill his vast mercantile enterprises, and finds time for outside operations of no small magnitude. Bennett was nearly seventy when he built the magnificent Herald buildings. Barnum is almost seventy, and his vigor and enterprise seem still to grow with his years.—Charles O'Connor, at the same age, is in the vigor of his powers. William B. Astor, over eighty, is always in his office down town at 9 o'clock, and never, even in stormy weather, though the distance is long, rides to and from his business. He has just finished his new residence in the 5th Avenue.

What does Mr. Emerson mean when he says, "Society never advances, that it recedes as fast on the one side as it gains on the other." Is it, if men will be intelligent and do brain-work and have character, they must lose in bodily powers and healthy functions? Must men be weaker, if they will be wiser? We do not believe a word of it. We do believe most cordially that character and mental activity prolong life; that Guizot, and Bryant, and Pius, and a multitude of others, are alive, because life in the spirit prolongs life in the flesh. Who does not know that the more intelligent interest and enthusiasm a man brings into his business, the more healthy, for this very reason, that business is for him.

There is a mysterious power in *interest* that cheats time and abates, nay, annihilates fatigue. How many times have I seen, when a boy upon a farm, a company of men move along at the haying and harvest, with a heavy step, and a languid swing of scythe and fork, and every little while some man more spiritless than the rest, cock his eye at the sun and guess the time of day ; how quickly have I seen all this change by the introduction of some stirring subject of conversation, in which all took part with animation. Again and again have I known them not only to move with a new life at their work, but work past the dinner hour without knowing it.

A medical gentleman told me that once when a young man, he started out very early in the morning from the city of Beyrout, with his brother, for a hunt in the foot hills of Lebanon. They walked fast and traveled far in search of game, but were continually disappointed. Late in the forenoon they reversed their steps, but they were yet far from home, when they began to feel so tired and exhausted that it seemed as though they should never reach home without a long rest; when, as they neared a large stream, some Arabs near by, observing their guns, told them there were wild ducks down in the rushes on the bank of that river. Instantly, said my friend, every particle of fatigue left us, we crept to the bank, shot and bagged our game, and went home in high spirits, and the distance seemed as nothing, now that we had our game to show.

A gentleman of somewhat sedentary habits, and withal a partial invalid, sometimes does a little surveying. He has often remarked that in cold weather, the men who carried chain and set stakes, who not only had the best of him in bodily vigor, but more exercise at the work, were always complaining of the cold, when he himself would scarcely feel the cold. The surveyor believed, as do I, that his feeling of interest and his brain-work, kept him warm and made this wonderful difference between him and his men.

A metropolitan physician, speaking of the remarkable good effects of high cheer upon the digestion, said that he once treated a dyspeptic, who invariably vomited his meals taken at a gloomy, up-town boarding house, and as invariably enjoyed and digested those he took down town with hilarious companions. How often has it happened that persons of even weak digestion, have taken rich suppers at parties, with lively companions and plenty of merriment, and felt no indigestion.

It is the brain that digests the food and makes a healthy liver, it is the brain that circulates the blood, it is the brain that breathes and walks, gives quality and energy to the voice and manner, wiriness and endurance to muscle. Anything less than this is to believe a man's body might go without a soul. How many thousand men have retired from the brain activities of a busy life, hoping for a long balance of years with a good time generally to the end, and have soon found that life had lost its inspiration, and they were fast coming to the end with dyspepsia and melancholy.

I knew well a clergyman who preached to ninety-four years and then died from accident. I knew another who preached to one hundred and two years. I knew a physician who practiced until seventy years of age and was then in perfect health. He dropped his practice and in two years died from dyspepsia and melancholy.

In how many thousand instances in our late civil war, did the man of intellect, the fine wrought scholar, the man whose body was tempered to his toes ends with character, wear out the man, and to the death, who was all brawn and no brains.

> " Know then that what'er of faith and cheer,
> Supports the soul, supports the body too."

A strong mind is full of hope, and hope is a far better tonic than *bark*. Courage resists disease, a great and absorbing purpose renews life, character, *cures* disease. Dr. Elder, who wrote his biography, once asked Dr. Kane for the best proved instance that he ever knew of the soul's power over the body—an instance that should push the hard-baked philosophy of materialism to the consciousness of its own idiocy. He paused a moment and then answered as with a spring, " The soul can lift the body out of its boots, sir. When our Captain was dying, I say *dying*—I have seen scurvy enough to know—I never saw a case so bad that either lived or died—there was trouble aboard, there might be mutiny. I went down to his bunk and shouted in his ear, ' mutiny ! Captain ! mutiny !' He shook off his cadaveric stupor. ' Set me up,' said he ; ' order these fellows before me !' He heard the complaint, ordered punishment, and from that hour recovered."

I once had under my professional care a young woman of fine constitution, clear head and energetic character. She had suffered long and severely from neuralgia, would often rise at night and walk the floor with the intensity of the pain. Her face, neck and shoulders were often swollen from no other cause than the recurring paroxyisms of this relentless malady. Medical treatment served only to palliate. I advised her to study medicine, partly hoping she might sometime achieve the profession, partly to test the power of a new and strong mental impression upon her disease. That women should become regular physicians, had then only been heard of in the case of the Blackwells, Mrs. Gleason, and one or two others. The project had for my patient the charm of novelty and heroism She took hold of the study with a most earnest and absorbing interest. The neuralgia waned, indeed, it seemed to retire as a guest forgotten and neglected, and was heard of no more.—My patient finished her course, kept her faith in herself, and became a successful practitioner in a metropolitan city. Her energy of purpose and her enthusiasm, made, as I believe, all the difference between cure and no cure in her case.

Another young woman came under my care having, as was supposed, a serious hip disease. The usual tortures of surgical counter-irritation had been gone through with, setons, moxas, etc., with no relief to the pain, irritation and lameness. She had not been long under treatment, before I had reason to believe the affection wholly hysterical. I had hardly time to act upon my new conviction, when I discovered my assistant—a woman of unusual sagacity—was already testing the effect of a new and strong mental impression. She began by saying to her, it is such a pity that you are *lame.* You must see if you do not get well how it must mar your prospects for life. Giving her a few days to grasp the full meaning of this, she further and plainly said, you must know that no young man could for a moment think of making you his wife, so helpless as you now are. Much more of this talk was given and received, in good faith, and the while my patient gained of her hip disease surprisingly, and in a few weeks returned home and engaged in active business. As a man or woman thinketh and believeth, so are they. The most painful cases I have ever had, were those invalids who never had any great, or noble, and absorbing purpose, or aim, or plan in life, and who could not be inspired with any. Their invalidism was a marvellous tissue of egoism and self-feeling.

The contrasts between barbarism and civilization, prove on a large scale, the influence of brains and character upon the physique and length of life. It has been fairly said that the New Zeland race is the most favorable type of barbarism in existence, having just emerged from an utterly savage condition. Cook and other navigators praise their robust physical aspect, and they are admitted to surpass our American Indians, as well as the finest African races. And how stands the account with these in comparison with English soldiers, and English students and men weighed at a Mechanics' Fair in Boston, and a company of West Point officers. The New Zelanders were in a sense picked men, being those in the employ of the English government.

The constant *average* height of the New Zelanders was less, and when compared with a class of eighty students at Cambridge, England, nearly two inches less. Their average weight was much less, their average area of chest was less, and the range of strength among the New Zelanders was from two hundred and fifty pounds to four hundred and twenty pounds, and among the soldiers from three hundred and fifty pounds to five hundred and four pounds!

But the test of longevity is after all the most decisive. The vast accumulation of facts and tables, bearing upon averages of life, made necessary by the immense business of life insurance, prove beyond cavil, that duration of life is a test of intelligence and civilization. In a single century the nations of Northern Europe, according to M. Quetelet, show a gain of ten to twenty-five per cent. In Geneva, Switzerland, where accurate registers have been kept for three hundred years, the average duration of life has in that time risen from twenty-one years to forty years. In Paris, five centuries ago, one in sixteen died annually, now one in thirty-two. In England, two centuries ago, one in thirty-three, now one in sixty. In half barbarous Russia, the mortality to-day is one in twenty-seven. I dare hazard the statement, imperfectly as I know the facts, that Cumming, and Gerhard, and Stanley, and Livingstone in Africa, and Lewis, Clarke and Fremont in our land, found no aborigines to compare with themselves in strength and endurance, or "*toughness*," in more homely English. The Indians, the best runners among them, could only equal Lewis' and Clarke's men, while to-day everywhere in our far western wilds, white men, herders and miners and ranchmen, are far more than a match for the brawny aborigines. In one case, Kit Carson and a band of trappers, six in all, fought two hundred savages a half-day, on the open plain, killing forty and routing the rest completely. When the savages came in sight on their fleet horses, a moment's deliberation, and our band of six killed every man his mule for a sort of breast work and to frighten the Indians' horses by the sight and smell of blood.

Do you remember that a young sprig of an artist once said, "Mr. Opie, pray sir, how *do* you mix your colors?" and the laconic reply, "with *brains* sir!" So I fancy I might have heard Kit Carson say, if asked how, without the loss of a man, he and his little band killed forty and routed the balance of two hundred savages, "with *brains*, sir!"

Finally, if we compare ancient nations among themselves, not one has ever survived conquest and removal from their own land but the Jews. And wherein did they greatly differ from other nations, but in that highest and best of all knowledge, the knowledge of the one only living and true God, his revealed will and the teachings of His Spirit. This knowledge, their love of religion, their faith in God and in self, gave to every Jew character and personality; wherein and wherefore they survive and are vigorous unto this day. The ten tribes were utterly lost, through the degradations of idolatry, followed by Syrian conquest.

Faith in God and in self-character, are our saviors. God is no respecter of *persons*, but of *character*.

